# Predictive Value of Clinical and CT Scan Findings for Complicated Appendicitis: A Retrospective Analysis

**DOI:** 10.7759/cureus.88948

**Published:** 2025-07-29

**Authors:** Ahmed G Alsayaf Alghamdi, Saleh M Alzhrani, Amer Fayraq, Saif A Alzahrani

**Affiliations:** 1 General Surgery, King Fahad General Hospital, Al Baha, SAU; 2 General and Colorectal Surgery, King Fahad General Hospital, Al Baha, SAU; 3 Preventive Medicine, King Abdullah International Medical Research Centre, Jeddah, SAU; 4 Preventive Medicine, King Abdulaziz Medical City Jeddah, Jeddah, SAU; 5 Preventive Medicine, Saudi Board of Preventive Medicine, Jeddah, SAU; 6 Public Health Agency, Ministry of Health, Riyadh, SAU; 7 Communicable Disease Prevention and Control, Public Health Authority, Riyadh, SAU

**Keywords:** alvarado score, appendectomy, cecal thickness, computed tomography (ct), ileum thickness, symptom duration

## Abstract

Background: Appendicitis is a common surgical emergency condition. Its timely and accurate diagnosis is crucial to prevent complications like perforation and abscess formation. However, distinguishing between uncomplicated and complicated appendicitis can be challenging. Clinical and imaging findings are often used, but their predictive value is still debatable. The objective of the study is to identify the predictive value of clinical and computed tomography (CT) scan findings for complicated appendicitis in patients who underwent appendectomy.

Methods: A retrospective analysis of data from a cohort of patients who underwent appendectomy in a tertiary governmental hospital was conducted. The study population included patients from varied demographic backgrounds, excluding those who were immunocompromised or had an appendectomy for reasons other than appendicitis. Data analysis was conducted using SPSS (IBM Corp., Armonk, NY, USA).

Results: Out of 256 patients, 57.4% were male and the majority were of Saudi origin (74.6%). A significant difference in the distribution of Alvarado scores between groups with complicated (6, IQR: 5-7) and uncomplicated appendicitis (5, IQR: 4-6) was observed. The logistic regression analysis revealed symptom duration, Alvarado score, and the presence of cecal and ileum thickness in CT scan as significant predictors for complicated appendicitis.

Conclusion: Symptom duration, Alvarado score, and the presence of cecal and ileum thickness in CT scan are significant predictors for complicated appendicitis. Healthcare professionals should consider these predictors when assessing patients with suspected appendicitis to facilitate early identification and management of potential complications. Further research is required to validate these predictors in different populations and settings.

## Introduction

Acute appendicitis represents one of the most common acute surgical emergencies worldwide [1]. The estimated prevalence of acute appendicitis is 90-100 cases per 100,000 individuals whereas lifetime incidence is around 7% [2]. Piotrowska et al. reported a complicated appendicitis incidence rate of 43.95% in a study involving 2,048 participants [3]. Despite the high prevalence of appendicitis, the diagnosis still remains a challenging area. Generally, clinical diagnosis is considered sufficient; however, 8-30% of the patients undergo unnecessary removal of the appendix [4]. Furthermore, it is crucial to discriminate uncomplicated appendicitis from complicated appendicitis because uncomplicated appendicitis can be treated using antibiotics instead of surgery [5,6]. Uncomplicated appendicitis is characterized by inflammation of the appendix with no signs of perforation and necrosis, while complicated appendicitis involves focal necrosis that leads to perforation [7].

Physical examination along with history taking is the first step in the diagnosis of appendicitis and is often reliable in most patients. Several tools have demonstrated efficacy in the diagnosis of appendicitis [8]. Clinical scoring systems and imaging techniques are mostly used to diagnose appendicitis [7]. The Alvarado score, a clinical scoring system, is the most popular among clinical scores despite its limitations among specific populations such as the elderly and pregnant [9]. Ultrasonography (US) is a non-invasive diagnostic approach that has been used for a long time. Some authors propose that the US should be the first imaging modality in the diagnosis of appendicitis as it decreases ionizing radiation exposure and the cost of the diagnostic procedure [10]. Magnetic resonance imaging (MRI) also provides an accurate diagnosis of appendicitis and is mostly used in children and pregnant patients [11].

Computed tomography (CT) is the gold standard method to diagnose appendicitis. It allows accurate localization of the appendix. Retroperitoneal space involvement is an important CT finding that predicts complicated appendicitis. Additional variables differentiating complicated from non-complicated appendicitis include the maximum appendiceal diameter, periappendiceal fat infiltration, and C-reactive protein levels [12]. Timely diagnosis of appendicitis is essential to prevent complications such as perforation and abscess formation. Moreover, distinguishing between complicated and uncomplicated appendicitis guides management strategies and helps avoid unnecessary surgical interventions. While clinical and imaging findings are commonly utilized for diagnosis, their predictive value remains controversial. Therefore, the current study aimed to identify the predictive value of clinical and CT scan findings for complicated appendicitis.

## Materials and methods

Study design

This retrospective study utilized previously collected clinical and CT findings in relation to complicated appendicitis. The definition of complicated appendicitis was based on intraoperative findings, with early-stage and suppurative appendicitis categorized as uncomplicated cases. Complicated appendicitis was identified by the presence of an abscess, mass, gangrene, or perforation.

Study setting

This study employed data from a retrospective cohort study focused on patients who underwent surgical intervention. The dataset encompassed patients with varied demographic characteristics who underwent either open laparotomy or laparoscopic surgery. All patients were sourced from a tertiary hospital that accepts both emergency room (ER) patients and those referred from other medical facilities. Data were originally collected from the surgical health records of the patients. An electronic instrument was designed to store the data of the selected sample. The collected data included patients' demographics, comorbidities, presenting symptoms, clinical findings, ordered imaging studies, computed tomography findings, and intraoperative findings. 

Study population

In this study, we considered all patients who had undergone an appendectomy procedure to be eligible for inclusion in our research. This encompassed a wide range of individuals from various backgrounds and health conditions. However, there were specific exclusions made in the interest of reducing confounding variables. Patients who were immunocompromised were excluded from the study. This included those who were currently receiving immunosuppression therapy or undergoing chemotherapy treatments, as well as patients who had been diagnosed with HIV. Additionally, patients who had an appendectomy for reasons other than appendicitis were also excluded. 

Statistical analysis

Data analysis was conducted employing SPSS version 29 (IBM Corp., Armonk, NY, USA). Continuous variables were examined for normality using the Kolmogorov-Smirnov test, which demonstrated a skewed distribution. These variables were expressed as minimum, maximum, median, and interquartile range (IQR). Categorical variables, on the other hand, were presented as frequencies and percentages. Alvarado scores were computed and classified as discharge (score ≤ 4), admission (score 5-6), and surgery (score ≥ 7). The outcome variable was defined as the presence of complication according to intraoperative findings, considered as a binary outcome. The Chi-square test was utilized to identify potential predictors, and when Chi-square assumptions were not met, the Exact test was applied. Binary logistic regression employing a forward conditional method was used to determine the most significant predictors for complicated appendicitis. A p-value of less than 0.05 was considered statistically significant.

Ethical considerations

This study was conducted in adherence to the principles of the Declaration of Helsinki and its later amendments. All patient data were anonymized and de-identified to maintain confidentiality. The retrospective nature of the study design meant that informed consent from patients was not obtained; however, all data was handled with utmost respect for patient privacy. Furthermore, all statistical analyses were performed in a manner that upheld the integrity of the research process and the reliability of the results.

## Results

The total sample consisted of 256 patients. The age ranged from seven to 91 years old, with a median of 29 years (IQR: 21.5-36). Of the total sample, 57.4% were male, and the majority were of Saudi origin (74.6%). When evaluating the distribution of comorbidities among the patient groups, 26.1% had diabetes mellitus, 15.2% had hypertension, and 13% had cardiovascular disease. The distribution of demographic characteristics and comorbidities among patient groups with complicated and uncomplicated appendicitis is further detailed in Table 1.

**Table 1:** Distribution of demographic characteristics and comorbidities among patient groups with complicated and uncomplicated appendicitis. *P-value calculated using Chi-square test ^P-value calculated using Fisher exact test n=256 DM: diabetes mellitus, HTN: hypertension, CVD: cardiovascular disease

Variable		Groups	Total	Complicated	Uncomplicated	Test statistics	P-value
Sex	Male		147 (57.4%)	26 (17.7%)	121 (82.3%)	0.413	0.610*
	Female		109 (42.6%)	16 (14.7%)	93 (85.3%)		
Nationality	Saudi		191 (74.6%)	25 (13.1%)	166 (86.9%)	6.04	0.014*
	Non-Saudi		65 (25.4%)	17 (26.2%)	48 (73.8%)		
Comorbidities	DM		12 (26.1%)	3 (25%)	9 (75%)	0.68	0.422*
	HTN		7 (15.2%)	3 (42.9%)	4 (57.1%)	3.67	0.089^
	CVD		6 (13%)	2 (33.3%)	4 (66.7%)	1.28	0.256^
	Coagulopathy		6 (13%)	1 (16.7%)	5 (83.3%)	<0.01	1.000^
	Other		25 (54.3%)	5 (20%)	20 (80%)	0.26	0.575^

**Figure 1:**
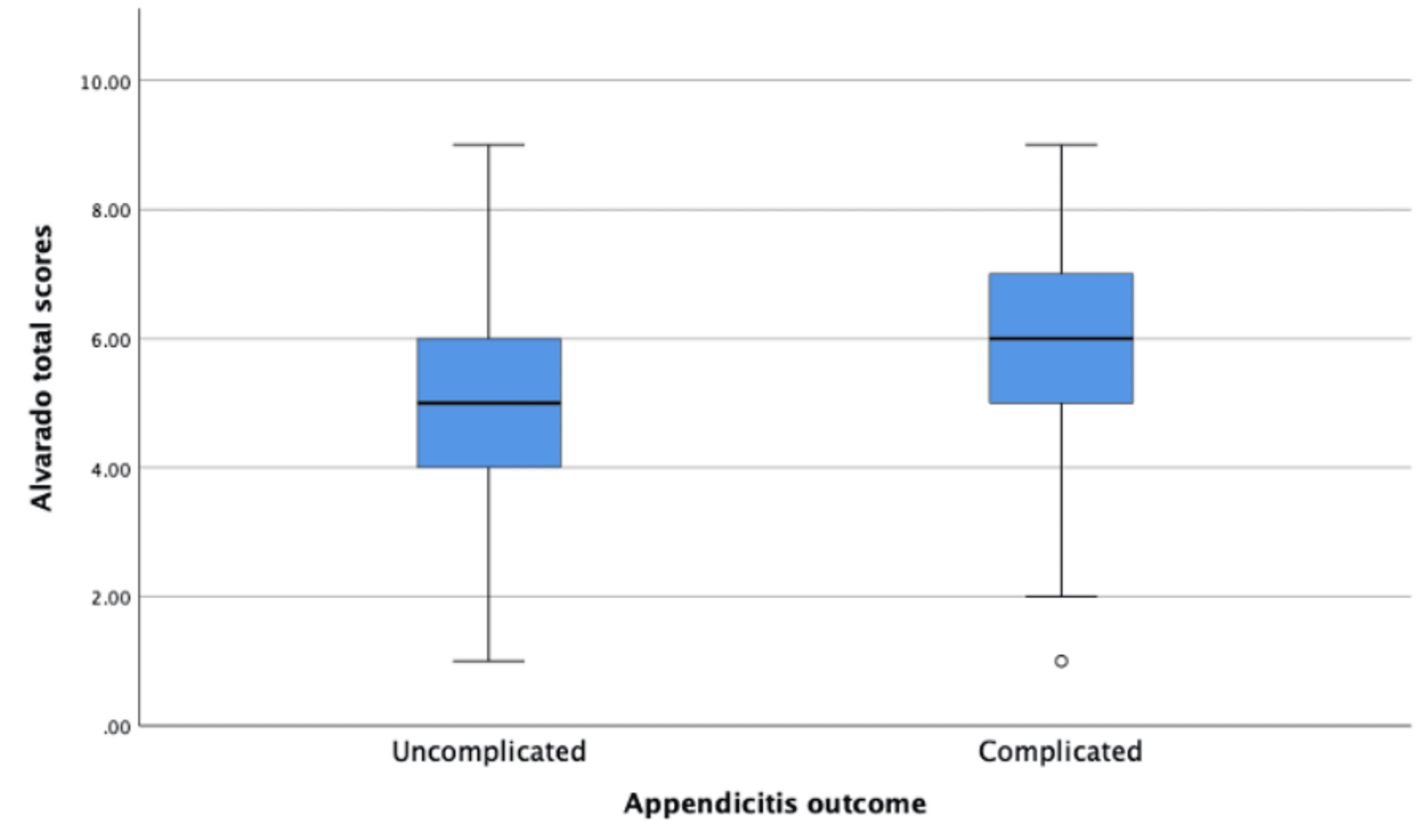
A boxplot showing the distribution of Alvarado scores among patients with complicated and uncomplicated appendicitis. n=256

Upon evaluation of the age among patients with complicated and uncomplicated appendicitis, there was no significant difference in the distribution of ages between the two groups (p=0.350). In the group with complicated appendicitis, the median age was 30 (IQR: 22-37), compared to those in the uncomplicated appendicitis group with a median of 28 (IQR: 21-34). Regarding Alvarado scores among patients, the median Alvarado score for the total patients was 5 (IQR: 4-6). While comparing complicated and uncomplicated appendicitis, we observed a significant difference in the distribution of scores between the two groups (p=0.015). In the group with complicated appendicitis, the median was 6 (IQR: 5-7), compared to uncomplicated appendicitis group which showed a median of 5 (IQR: 4-6). Further details of the distribution of Alvarado scores can be observed in Figure 1.

In the assessment of symptom duration, 49.6% of patients had onset of symptoms within 24 hours and the remaining 50.4% reporting onset before 24 hours. Upon comparing the complicated and uncomplicated appendicitis groups, significant differences were evident. The onset of symptoms of less than 24 hours was associated with a lower proportion of complicated appendicitis cases (9.4%) compared to those with symptom onset after 24 hours (23.3%) (p=0.004). As for the Alvarado scores, 38.7% of patients were recommended for discharge, 36.7% for admission, and 24.6% for surgery. CT scans were ordered for 69.1% of patients, while 30.9% did not have CT scan. The distribution of Alvarado scores varied significantly between the groups, with a higher proportion of patients categorized for surgery (28.6%) having complicated appendicitis compared to those advised for discharge (9.1%) or admission (16%) (p=0.005). See the detailed results in Table 2.

**Table 2:** Distribution of symptom duration, Alvarado score, and CT scan in patient groups with complicated and uncomplicated appendicitis *P-value calculated using Chi-square test ^P-value calculated using Fisher exact test n=256

Variable	Groups	Total	Complicated	Uncomplicated	Test statistics	P-value
Duration of symptoms	WIthin 24 hours	127 (49.6%)	12 (9.4%)	115 (90.6%)	8.89	0.004*
	After 24 hours	129 (50.4%)	30 (23.3%)	99 (76.7%)		
Alvarado score	Discharge	99 (38.7%)	9 (9.1%)	90 (90.9%)	10.66	0.005*
	Admission	94 (36.7%)	15 (16%)	79 (84%)		
	Surgery	63 (24.6%)	18 (28.6%)	45 (71.4%)		
Patient undergone CT scan	Yes	177 (69.1%)	28 (15.8%)	149 (84.2%)	0.04	0.858^
	No	79 (30.9%)	14 (17.7%)	65 (82.3%)		

As per the CT scan findings detailed in Table 3, fluid around the appendix was seen in 33.9% of patients, with 23.3% of such cases being complicated appendicitis (p-value=0.050). Lymph node enlargement was detected in 40.1% of patients, and among these, 22.5% were complicated appendicitis cases (p-value=0.045). Fat stranding was present in 66.7% of patients, with 17.8% of these cases being complicated appendicitis (p-value=0.308). Cecal thickness was noted in 14.1% of patients, with a higher complication rate of 36% (p-value=0.006). Ileum thickness was identified in 7.9% of patients and showed a 50% complication rate (p-value=0.002). In 9% of patients where the appendix was not visible, a complication rate of 12.5% was observed (p-value=0.752). Lastly, 3.4% of patients presented with unremarkable CT scan findings, and none of these patients had any complications (p-value=0.592). Further details regarding CT scan findings are shown in Table 3.

**Table 3:** Distribution of CT scan findings in patient groups with complicated and uncomplicated appendicitis, including odds ratio (OR) values *P-value calculated using Chi-square test ^P-value calculated using Fisher exact test n=256

Variable	Groups	Total	Complicated	Uncomplicated	OR	Test statistics	P-value
Fluid around appendix	Yes	60 (33.9%)	14 (23.3%)	46 (76.7%)	2.2	3.85	0.050*
	No	117 (66.1%)	14 (12%)	103 (88%)			
Lymph node enlargement	Yes	71 (40.1%)	16 (22.5%)	55 (77.5%)	2.3	4.01	0.045*
	No	106 (59.9%)	12 (11.3%)	94 (88.7%)			
Fat stranding	Yes	118 (66.7%)	21 (17.8%)	97 (82.2%)	1.6	1.04	0.308*
	No	59 (33.3%)	7 (11.9%)	52 (88.1%)			
Fecalith	Yes	14 (7.9%)	2 (14.3%)	12 (85.7%)	0.88	0.03	1.000^
	No	163 (92.1%)	26 (16%)	137 (84%)			
Cecal thickness	Yes	25 (14.1%)	9 (36%)	16 (64%)	3.9	8.9	0.006^
	No	152 (85.9%)	19 (12.5%)	133 (87.5%)			
Ileum thickness	Yes	14 (7.9%)	7 (50%)	7 (50%)	6.8	13.34	0.002^
	No	163 (92.1%)	21 (12.9%)	142 (87.1%)			
Appendix not seen	Yes	16 (9%)	2 (12.5%)	14 (87.5%)	0.74	0.15	0.752^
	No	161 (91%)	26 (16.1%)	135 (83.9%)			
Unremarkable	Yes	6 (3.4%)	0 (0%)	6 (100%)	0.84	1.17	0.592^
	No	171 (96.6%)	28 (16.4%)	143 (83.6%)			

In the logistic regression analysis, the duration of symptoms, Alvarado score, and the presence of cecal and ileum thickness in CT scan findings were significant predictors for complicated appendicitis. The symptom duration had an odds ratio (OR) of 2.83, indicating that patients with complicated appendicitis had about 2.83 times higher odds of symptom onset greater than 24 hours. The Alvarado score had an OR of 1.339. While the presence of cecal and ileum thickness in CT scan had an OR of 11.054, suggesting that complicated appendicitis cases have 11 times the odds of having cecal and ileum thickness in their CT. The detailed logistic regression analysis is shown in Table 4.

**Table 4:** Logistic regression analysis showing the predictors for complicated appendicitis, including symptom duration, Alvarado score, and CT findings (R2=0.099, model significance =<0.001) n=177

	B	OR (95% CI)	P-value
Constant	-4.99	0.007	< .001>
Duration of symptoms	1.04	2.8 (1.3-6)	0.007
Alvarado.total	0.292	1.3 (1.1-1.6)	0.005
Cecal thickness (CT) by Ileum thickness (CT)	2.403	11.1 (2.4-50.4)	0.002

## Discussion

The present study aimed to find the predictive values of clinical and CT scan findings for complicated appendicitis. The findings of the present study showed that the duration of symptoms, Alvarado score, and cecal and ileum thickness in CT scans were the main predictors of complicated appendicitis, with the highest OR reported for cecal and ileum thickness in CT. Overall, our results showed that the combination of clinical and imaging features can help in the accurate diagnosis of complicated appendicitis. Bom et al., in their narrative review, also reported that incorporating imaging with clinical scores can help in differentiation between complicated and uncomplicated appendicitis [7].

The duration of symptoms was identified as a significant factor that differs between uncomplicated and complicated appendicitis. Our results showed that complicated appendicitis has about 2.83 times higher odds of symptom onset greater than 24 hours compared to uncomplicated appendicitis. In complicated appendicitis, symptoms can extend beyond 48 hours. However, in approximately 75% of the cases, patients present within 24 hours after the onset of the symptoms [13]. These findings have also been supported by a study carried out by Lietzen et al. to assess the possibility of differentiating complicated appendicitis from uncomplicated appendicitis without imaging. The results of their study showed that symptom duration is a significant factor in identifying the type of appendicitis in patients [14]. As far as the age distribution of the patients is concerned, no major difference was observed between the groups of patients. It suggests that the presence of uncomplicated and complicated appendicitis is independent of age. However, these findings are different from the results of a study conducted by Kim et al. They suggested that there was a difference in mean age among the patients with uncomplicated and complicated appendicitis. The mean age was higher in the individuals having complicated appendicitis [12].

Alvarado scores help in predicting the occurrence of complicated appendicitis. In the current study, a significant difference was observed in Alvarado scores between complicated and uncomplicated groups of patients. The median scores were greater in patients with complicated appendicitis as compared to patients with uncomplicated appendicitis. Our results are not in accordance with a previous study conducted by Deiters et al. in which they investigated whether Alvarado scores differ significantly among patients with uncomplicated and complicated appendicitis [15]. Their findings showed that there was no considerable difference in mean Alvarado scores among the patients with uncomplicated and complicated appendicitis [15]. Similar results were also obtained in a study conducted by Haak et al. They investigated the effectiveness of Alvarado scores in distinguishing between both types of appendicitis. They suggested that these scores have limited capability to differentiate between uncomplicated and complicated appendicitis [16].

Our results demonstrated that CT scan findings are very good at predicting the presence of complicated appendicitis. A significant difference in CT scan findings was found in patients having uncomplicated and complicated appendicitis. Similar results have been reported in a systematic review and meta-analysis conducted by Kim et al. [17]. They studied various features of CT scan including extraluminal air, ascites, ileus, intraluminal appendicolith, ileus, abscess, periappendiceal fluid, and appendiceal wall enhancement defect and concluded that these are significant in distinguishing uncomplicated appendicitis from complicated appendicitis [17]. Similarly, Leitzen et al. also concluded that CT imaging should be utilized for the differential diagnosis of complicated and uncomplicated acute appendicitis [14]. In their study, they reported that clinical findings and laboratory biomarkers cannot alone predict the severity of acute appendicitis [14]. The findings of the current study are also in line with a systematic review conducted by Bom et al. They assessed the effectiveness of MRI, ultrasound imaging, and CT scans for differentiating between both types of appendicitis. They concluded that these diagnostic modalities are limited in their capability to differentiate between uncomplicated and complicated appendicitis. However, a CT scan is sensitive and specific in identifying the difference between the two types of appendicitis [18]. Pickhardt et al. also resonated with these findings as they suggested that multidetector CT is a reliable method for accurately diagnosing acute appendicitis [19].

Iamwat et al. conducted a retrospective investigation of 201 patients who underwent appendectomy. All patients underwent CT imaging prior to appendectomy. Their findings showed that CT imaging had a sensitivity of 87.2% for the differential diagnosis of complicated from uncomplicated acute appendicitis, whereas specificity and accuracy were 75.7% and 81.1%, respectively [2].

## Conclusions

Our findings indicate that symptom duration, Alvarado score, and the presence of cecal and ileum thickness in CT scan are significant predictors for complicated appendicitis. Patients with symptom onset greater than 24 hours and those with cecal and ileum thickness in their CT scans are at a higher risk of developing complicated appendicitis. Therefore, these factors should be taken into consideration when evaluating patients with suspected appendicitis in order to facilitate the early identification and management of potential complications. We recommend that healthcare professionals consider these predictors when making their initial assessment of patients presenting with symptoms suggestive of appendicitis. This could guide the decision for early surgical intervention, especially in patients with symptom onset greater than 24 hours and the presence of cecal and ileum thickness in their CT scans. Further research is required to validate these predictors in different populations and settings.
